# Genome-wide expression and response to exposure-based psychological therapy for anxiety disorders

**DOI:** 10.1038/tp.2017.177

**Published:** 2017-08-29

**Authors:** S Roberts, C C Y Wong, G Breen, J R I Coleman, S De Jong, P Jöhren, R Keers, C Curtis, S H Lee, J Margraf, S Schneider, T Teismann, A Wannemüller, K J Lester, T C Eley

**Affiliations:** 1 King’s College London, Social, Genetic and Developmental Psychiatry Centre, Institute of Psychiatry, Psychology and Neuroscience, London, UK; 2 National Institute for Health Research Biomedical Research Centre, South London and Maudsley National Health Service Trust, London, UK; 3 Dental Clinic Bochum, Bochum, Germany; 4 School of Biological and Chemical Sciences, Queen Mary University of London, London, UK; 5 Mental Health Research and Treatment Center, Ruhr-Universität Bochum, Bochum, Germany; 6 School of Psychology, University of Sussex, Brighton, UK

## Abstract

Exposure-based psychological treatments for anxiety have high efficacy. However, a substantial proportion of patients do not respond to therapy. Research examining the potential biological underpinnings of therapy response is still in its infancy, and most studies have focussed on candidate genes. To our knowledge, this study represents the first investigation of genome-wide expression profiles with respect to treatment outcome. Participants (*n*=102) with panic disorder or specific phobia received exposure-based cognitive behavioural therapy. Treatment outcome was defined as percentage reduction from baseline in clinician-rated severity of their primary anxiety diagnosis at post treatment and 6 month follow-up. Gene expression was determined from whole blood samples at three time points using the Illumina HT-12v4 BeadChip microarray. Linear regression models tested the association between treatment outcome and changes in gene expression from pre-treatment to post treatment, and pre-treatment to follow-up. Network analysis was conducted using weighted gene co-expression network analysis, and change in the detected modules from pre-treatment to post treatment and follow-up was tested for association with treatment outcome. No changes in gene expression were significantly associated with treatment outcomes when correcting for multiple testing (*q*<0.05), although a small number of genes showed a suggestive association with treatment outcome (*q*<0.5, *n*=20). Network analysis showed no association between treatment outcome and change in gene expression for any module. We report suggestive evidence for the role of a small number of genes in treatment outcome. Although preliminary, these findings contribute to a growing body of research suggesting that response to psychological therapies may be associated with changes at a biological level.

## Introduction

Anxiety disorders are some of the most common mental health disorders, affecting a sizable proportion of the population.^1^ It is estimated that over 25% of people will be diagnosed with an anxiety disorder at some point during their lifetime, reflecting a considerable economic cost to society.^1, 2^ In addition to the emotional discomfort and distress central to the diagnosis, anxiety disorders are associated with numerous negative consequences for many areas of everyday functioning.^3^ Psychological therapies such as cognitive behavioural therapy (CBT) and exposure therapy in particular have a relatively high efficacy for treating anxiety.^4, 5, 6^ Meta-analyses have demonstrated large effect sizes for a range of anxiety diagnoses, and treatment response shows substantial continuity across long-term outcomes.^7, 8^

Therapygenetics is a relatively new area of research that investigates genetic predictors of response to psychological therapies.^9^ Most studies have focussed on candidate gene predictors of treatment response, although results have been inconsistent.^10^ In addition, recent preliminary research has identified epigenetic changes in genes of interest during psychological therapies that may be associated with outcome. Studies thus far have focussed on *BDNF* in adults with borderline personality disorder,^11^ *SERT* in children with anxiety disorders,^12^ *MAOA* in adults with panic disorder^13^ and the HPA-axis-related genes *FKBP5* and *GR* in children with anxiety disorders^14^ and in veterans with posttraumatic stress disorder.^15^ Interestingly, in the latter study, changes in *FKBP5* methylation during exposure therapy were found to be associated with *FKBP5* expression at follow-up.

A small number of further studies have examined the role of gene expression and response to a psychological therapy. Gene expression levels are a useful indicator of gene activity and function as they are known to be highly dynamic. They are also susceptible to genetic, epigenetic and environmental influences, and therefore may provide insight into the potential mechanisms of therapy response. Two studies have demonstrated that *FKBP5* expression may be associated with outcome following CBT in participants with posttraumatic stress disorder, with both finding that increases in gene expression during therapy were associated with symptom improvement.^16, 17^ Another two studies have taken a broader approach, assessing gene expression levels of panels of genes in major depressive disorder before and after CBT.^18, 19^ In the first of these studies, 10 genes were assayed before and after treatment using the BioM-10 panel, which consists of five genes associated with high mood states and five genes associated with low mood states.^18^ Clinical improvement after CBT was associated with an increase in the high mood markers relative to the low mood markers. The second study identified gene expression networks predictive of remission, whereby significant co-expression networks of genes before treatment were identified in remitters but not in patients who remained depressed.^19^ Although preliminary, these studies suggest that gene expression levels may be indicative of response to treatment.

To date, no studies have utilised a genome-wide approach to study whether gene expression changes are associated with response to a psychological therapy. Such hypothesis-free analysis allows for the testing of a wider range of novel genomic targets, including rare variants, as well as the opportunity to identify networks of genes with similar activity. Here we assess gene expression levels across the genome to examine transcriptomic changes that may be associated with response to psychological therapy. We examined the association between clinical symptomatology and change in probe-level gene expression from pre-treatment to post treatment and follow-up. Biological pathway enrichment analysis was performed using the ranked gene list, in order to identify known pathways and functions represented in the top results. Further data-driven clustering techniques were used to identify networks of co-expressed genes in the data set, and the trajectory of these modules across the course of treatment was examined. Co-expression network analysis using weighted gene co-expression network analysis (WGCNA) is a powerful approach, even in relatively small data sets, as it describes the correlation patterns among genes and therefore reduces the multiple testing burden associated with genome-wide data. This is the largest study of gene expression and treatment response, and, to our knowledge, the first to include transcriptomic data at follow-up as well as pre- and post treatment.

## Materials and methods

### Participants

Participants (*n*=102) were recruited at the Mental Health Research and Treatment Center, Ruhr-Universität Bochum, Germany (*n*=56) or the Dental Clinical Bochum, Germany (*n*=46). Age at baseline ranged from 19 to 68 years (mean=39.8), and 66.7% of the sample were female. At baseline, 27.5% were smokers, 5.9% were using a form of psychoactive medication, and 32.4% took other regular medications. All participants were treated for panic disorder with agoraphobia, or agoraphobia alone (28.4%) or specific phobia (71.6% including dental fear—45.1% of total). Diagnoses were made according to DSM-IV criteria by trained clinicians using the Diagnostisches Interview bei Psychischen Störungen (DIPS) and Mini-DIPS,^20, 21, 22^ structured interviews with well-established reliability, validity and patient acceptance.^23, 24, 25, 26^ At least one comorbid diagnosis was present in 38.2% of participants. All participants completed one of four exposure therapy or exposure-based CBT treatment programmes as detailed below. A diagram of treatment protocols can be found in Supplementary Figure S1. All treatments were regularly supervised by experienced senior clinicians using audiovisual recordings in order to ensure treatment protocol integrity.

Treatment was administered at the Mental Health Research and Treatment Centre in three groups. All participants received five preliminary sessions covering diagnosis and psychoeducation before starting therapy. Participants with a primary diagnosis of panic disorder/agoraphobia were randomized either to exposure-based CBT (akin to the specific phobia group above) or to an exposure-alone condition without any element of cognitive restructuring (Clinical Trials: NCT01680327). Participants with specific phobia (not primarily associated with dental fear) were treated in a long-term programme of up to 25 sessions of *in vivo* exposure. Participants in these groups were excluded if they were using anxiolytic medication.

Individuals with high levels of dental fear were treated in a dental anxiety-specific programme.^27^ Treatment was given in five sessions, including an initial diagnostic and psychoeducation session, and a session developing relaxation techniques and focussing on helpful thoughts. These coping strategies were then encouraged in three sessions consisting of exposure scenarios such as video exposure, noise exposure and *in sensu* exposure (virtual reality or visualisation). Concurrent psychoactive medication was not an exclusion criterion.

### Outcome measures

#### Severity—clinical global impression

Treatment response was defined as percentage improvement in clinician-rated severity of the treated diagnosis, as determined using the Clinical Global Impression—Severity (CGI-S) scale.^28^ The CGI-S consists of a scale of 1–7, with a score of 1 indicating that the patient is healthy, and 7 indicating that the patient is extremely ill. Mean CGI-S score at baseline was 4.46 (s.d.=1.14), signifying that the sample was moderate to markedly ill. Scores were rescaled from 1–7 to 0–6 and percentage improvement from pre-treatment to post treatment and pre-treatment to follow-up was calculated for all participants.

### Ethics statements

This study was conducted in accordance with the principles outlined by the Declaration of Helsinki. Site-specific trials and the collection of samples were approved by local Human Ethics and Biosafety Committees, and all participants provided informed consent. The receipt, storage and analyses of samples were approved by the London-Bentham NRES Committee and the King’s College London Psychiatry, Nursing and Midwifery Research Ethics Sub-Committee.

### Genome-wide expression data

#### Sample collection

Whole blood samples were drawn at pre-treatment (before exposure), immediately post treatment and follow-up (~6 months following the conclusion of treatment) using PAXgene blood RNA tubes. Blood RNA was isolated and purified using the PAXgene Blood miRNA Kit (Qiagen, Hilden, Germany) according to the manufacturer’s protocol using the Qiagen Qiacube. RNA quality and integrity were measured using both the Nanodrop 1000 spectrophotometer (Nanodrop 1000, Nanodrop, Wilmington, DE, USA) and the Agilent 2100 Bioanalyzer (Agilent, Santa Clara, CA, USA). Genome-wide expression levels were measured from 750 ng total RNA using the Illumina HumanHT-12v4 Expression BeadChip (Illumina, San Diego, CA, USA).

#### Quality control

Initial processing of data was performed using GenomeStudio to identify samples with detection rates dissimilar to the rest of the project (GenomeStudio, Illumina). Stringent quality control processing of the data was conducted using the standardised procedures from internal pipelines (available at https://github.com/snewhouse/BRC_MH_Bioinformatics). Expression data were background-corrected using module-based background correction for Beadarray.^29^ Probes were then filtered by selecting probes with expression levels >2 s.d. greater than the mean intensity of the negative control (background) beads. Reported gender was compared to *XIST* gene expression (female specific) and Y chromosome gene expression (male specific). Each probe was then transformed and normalised using log2 transformation and robust-spline normalisation.^30, 31^ Sample relationships within the co-expression network were assessed and outliers were removed.^32^ Following sample outlier removal, 95 samples at pre-treatment, 99 samples at post treatment and 97 samples at follow-up remained. Probes detected in <80% of the sample were removed. Following quality control procedures, 4381 probes with high-quality data were available for analysis. All quality control was performed in R^33^ making use of the lumi package.^31^

#### Batch effects

In order to minimise potential batch effects, all three time points per participant were extracted in the same batch and run on the same array. Technical batch variables (hybridisation, sample position on microarray, RNA integrity number (RIN), overall processing batch, date of RNA extraction, date of amplification, date of microarray processing and cRNA concentration) were assessed for association with the first principal component of the expression data using stepwide linear regression bootstrapped 100 times (with covariate order randomised). The data were then adjusted for the associated batch variables. ComBat (from the sva package) was used to control for hybridisation (expression microarray, 27 chips), with fixed effects of RIN and cRNA concentration.^34^ The data were then adjusted for RIN and cRNA concentration using multivariate linear regressions (RcppArmadillo).^35^ Nine technical replicates (three participant sample sets) were included as quality checks. Duplicate samples showed very high consistency, with an average concordance of *r*=0.986.

#### Cell-type composition

Cell-type proportions (lymphocyte, neutrophils and monocytes) were estimated using deconvolution methods implemented in CellMix^36^ based on previously reported whole blood cell composition values.^37^ Change in lymphocyte, neutrophil and monocyte proportions were calculated for pre- to post treatment, and pre-treatment to follow-up.

### Analyses

#### Probe level analyses

Change in expression from pre- to post treatment and pre-treatment to follow-up were calculated for all probes (total *n*=4381) after quality control.

#### Confounding factors

Age, body mass index, gender, comorbidities, smoking status, psychoactive medication, other medications and cell-type composition changes were tested for association with outcome. Population stratification was tested for association with outcome using the first two principal components from genome-wide genotyping data (‘PC1’, ‘PC2’).^38^ Treated diagnosis was not associated with outcome at post treatment (*F*=0.44, *P*=0.644) or at follow-up (*F*=0.74, *P*=0.480).

#### Gene expression and treatment outcome

The association between change in expression values and percentage reduction in CGI-S was tested using linear regression models for post treatment and follow-up. Time (number of days since pre-treatment) and number of sessions were included as covariates to account for variability in treatment length as well as baseline CGI-S and psychoactive medication status. Robust standard errors and clustering by treatment group were used to account for differences between treatment conditions. In these analyses, a negative *β-*value indicates that a greater percentage reduction in severity is associated with a decrease in gene expression over time. Linear regression analyses were conducted in Stata.^39^

#### Functional annotation and enrichment analysis

Illumina microarray probes were annotated using the Bioconductor package lumiHumanIDmapping. Probes were ranked according to linear regression analysis uncorrected *P*-value, and assessed for enrichment of biological pathways using GOrilla.^40^ Where probes mapped to the same gene, the highest-ranked was retained. This method performs Gene Ontology (GO) enrichment analyses on ranked probe lists to identify known pathways more strongly associated with the outcome of interest. Further information on this analysis is provided in the Supplementary Information.

#### Multiple testing correction

The *P*-values for probe-wise analyses were corrected for multiple testing using the method defined by Benjamini and Hochberg^41^ to give a test *q*-value. False discovery rate statistics (FDR; *q*-values) were calculated using the qvalue package in R. Probes associated with treatment outcome with a FDR-corrected *q*<0.05 were considered significant. Probes associated with treatment outcome with a FDR-corrected *q*<0.5 are reported as suggestive.

### Network-based analyses

Data-driven clustering was performed using weighted gene co-expression network analysis on pre-treatment samples to create signed co-expression networks (WGCNA).^42^ Modules detected at pre-treatment were forced onto the data at the post treatment and follow-up time points using the module preservation function^43^ in order to examine changes across the course of treatment.

Module eigengenes, equivalent to the first principal component of each module, were used as a proxy for module expression. The association between change in CGI-S and change in module expression was tested using linear regression models for post treatment and follow-up as previously described. The grey module was not included in any analyses as it consisted of genes that were unable to be assigned to a module.

### Code and data availability

Code used for data QC can be accessed at https://github.com/snewhouse/BRC_MH_Bioinformatics. All analysis scripts are available on request. The data discussed in this publication have been deposited in NCBI’s Gene Expression Omnibus^44^ and are accessible through GEO Series accession number GSE94119 (https://www.ncbi.nlm.nih.gov/geo/query/acc.cgi?acc=GSE94119).

## Results

### Clinical characteristics

As expected, there was a significant change in CGI-S from pre-treatment to post treatment (pre-treatment=4.46, post treatment=2.04; *t*=17.831, df=99, *P*<0.001) with a mean percentage change in severity of 53.14%. There was also a significant change in CGI-S from pre-treatment to follow-up (follow-up=2.36; *t*=12.140, df=93, *P*<0.001) where the mean percentage change in severity was 45.80%.

### Confounding factors

There was no association between treatment outcome and baseline severity, age, body mass index, gender, comorbidities, smoking status, other (non-psychoactive) medications, cell-type proportion changes and population stratification values. However, a significant association was reported between psychoactive medication and percentage reduction in CGI-S from pre- to post treatment and follow-up (Table 1—participants on psychoactive medications showed a poorer response to treatment), and was therefore included as a covariate in linear regression analyses.

### Probe level analyses

#### Post treatment

**Figure 1:**
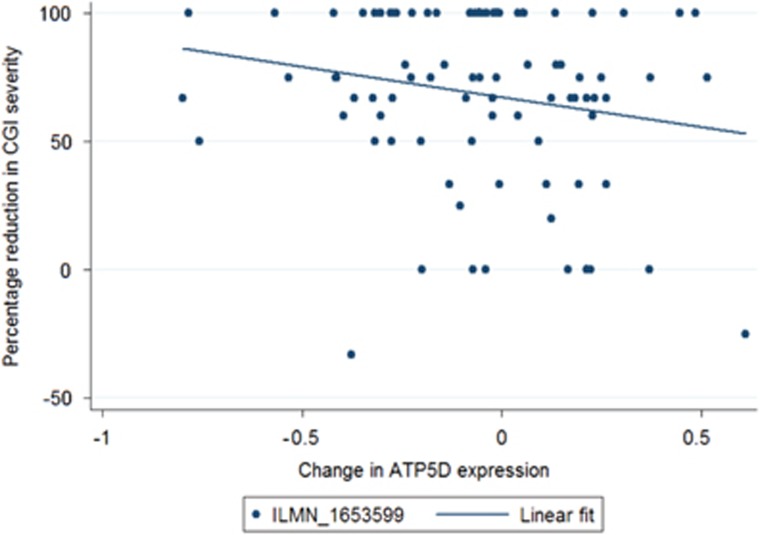
Change in gene expression and percentage reduction in Clinical Global Impression—Severity (CGI-S) at post treatment. *ATP5D*; *β*=−0.373, *P*=8.90E−05.

Of the 4381 probes tested, 171 showed a nominally significant association between changes in expression and percentage improvement in symptoms from pre-treatment to the post-treatment time point (*P*<0.05). Table 2 shows the top 10 probes ranked by uncorrected *P*-value including standardised test statistics. No probes showed a significant association with treatment outcome when corrected for multiple testing (*q*<0.05). One probe reached a suggestive threshold (*q*<0.5); ILMN_1653599 (Figure 1, *q*=0.38, *P*=8.90 × 10^−5^). Annotation showed that this probe mapped to the gene *ATP5D*. Further top-ranked probes are detailed in Supplementary Table S1.

#### Enrichment analyses

Enrichment analysis of the ranked probes from the post-treatment analyses using GOrilla yielded nine significant GO terms at *q*<0.05, with some terms depicting related functions. The top reported pathway was ‘establishment of protein localisation to endoplasmic reticulum’ (*P*=3.51 × 10^−6^, *q*=0.03). A full list of pathways can be found in Supplementary Table S2.

#### Follow-up

**Figure 2:**
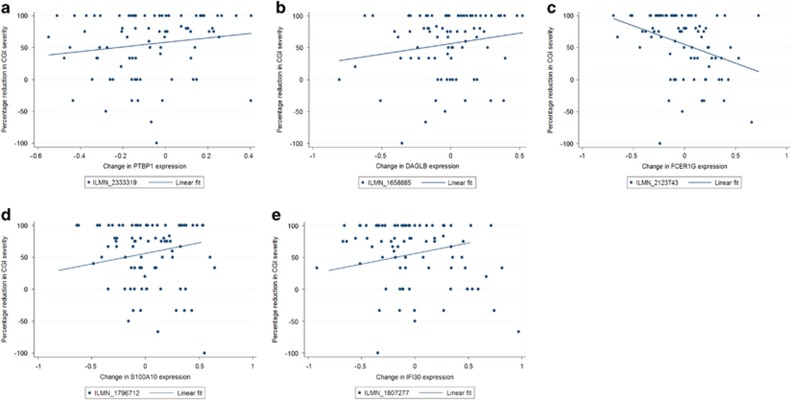
Change in gene expression and percentage reduction in Clinical Global Impression—Severity (CGI-S) at follow-up for five gene-labelled probes significant at a suggestive level. (**a**) *PTBP1*; *β*=0.715, *P*=4.11E−05. (**b**) *DAGLB*; *β*=0.713, *P*=6.02E−04. (**c**) *FCER1G*; *β*=−1.176, *P*=6.33E−04. (**d**) *S100A10*; *β*=−0.428, *P*=1.24E−03. (**e**) *IFI30*; *β*=*−*0.677, *P*=1.12E−03.

In total, 225 probes showed a nominally significant association between changes in expression and percentage improvement in symptoms from pre-treatment to the follow-up time point. Table 2 shows the top 20 probes ranked by uncorrected *P*-value including standardised test statistics. Similarly to the findings for the post-treatment analyses, no probes showed a significant association when correcting for multiple testing. However, 19 probes reached a suggestive threshold (Table 2). Figure 2 shows the top five gene-labelled probes; ILMN_2333319 (*q*=0.49, *P*=2.01 × 10^−4^), ILMN_1658885 (*q*=0.49, *P*=4.33 × 10^−4^), ILMN_2123743 (*q*=0.49, *P*=5.73 × 10^−4^), ILMN_1807277 (*q*=0.49, *P*=1.12 × 10^−3^) and ILMN_1796712 (*q*=0.49, *P*=1.24 × 10^−3^). These probes corresponded with the genes *PTBP1, DAGLB, FCER1G, IFI30* and *S100A10*. Further top-ranked probes are detailed in Supplementary Table S3.

#### Enrichment analyses

Enrichment analysis of the ranked probes from the follow-up analyses using GOrilla yielded a number of significant GO terms at *q*<0.05, with many terms depicting processes related to regulation of cell cycle pathways. The top-reported pathway was ‘negative regulation of apoptotic process’ (*P*=5.07 × 10^−6^, *q*=0.043). A full list of identified pathways can be found in Supplementary Table S4.

### Network-based analyses

Six co-expression modules were detected at pre-treatment (excluding the grey module). Modules were well preserved at post treatment and follow-up, indicating little change in the composition of modules across the treatment time points (module details can be found in the Supplementary Information). No significant association was found between percentage reduction in CGI-S and change in module eigengene for any module at post treatment or follow-up (result tables provided in Supplementary Information).

## Discussion

To our knowledge, this is the first study to examine genome-wide gene expression profiles and psychological treatment response. We examined the relationship between transcriptomic changes across the full course of treatment (pre-treatment, post treatment and follow-up) and treatment outcome in 102 adults receiving exposure-based CBT. Although no probes reached significance when correcting for multiple testing (*q*<0.05), expression changes in a small number of probes were associated with changes in symptom severity from pre-treatment to post treatment and pre-treatment to follow-up at a suggestive level (*q*<0.5).

In the post-treatment analyses, the top nominally significant probe was in a gene related to mitochondrial ATP synthase (*ATP5D*). A further related gene (*ATP5H*) was also within the top probes. The function of these genes is moderately well characterised. Recently, it has been suggested that mitochondrial dysfunction may play a role in disorders such as schizophrenia^45^ although the importance of these processes for mental health outcomes such as anxiety and depression is not clear.

A small number of interesting probes reached a suggestive threshold in the follow-up analyses. The top nominally significant probe was related to the gene *PTBP1,* which plays a functional role in pre-mRNA processing and the regulation of splicing events. Recent research has suggested that downregulation of the gene may be a risk factor for Parkinson’s disease,^46^ however there is little evidence as yet for the role of the gene in psychiatric outcomes.

A key probe of interest at follow-up was part of the diacylglycerol lipase beta gene (*DAGLB*). *DAGLB* is known to be involved in the biosynthesis of 2-arachidonoyl-glycerol (2-AG), a key endogenous endocannabinoid in the endocannabinoid signalling system.^47, 48^ Interestingly, the role of the endocannabinoid system in anxiety and anxious behaviours is supported by a considerable body of research in both human and animal studies.^49, 50, 51^ In this study, we found an increase in DAGLB expression with greater treatment response. Previous research has demonstrated that reduced 2-AG levels are associated with increased risk of outcomes such as post-traumatic stress disorder and major depression,^52, 53, 54^ while animal studies have demonstrated that increases in 2-AG through pharmacological intervention may reduce anxiety-like behaviours.^55, 56^ Given the role of *DAGLB* in the synthesis of 2-AG, it is therefore of great interest that increases in *DAGLB* expression corresponded with greater reductions in severity, while decreases in *DAGLB* expression corresponded with lower reductions in severity. Genetic variation in the endocannabinoid system has also been investigated with respect to psychological therapy response, whereby several polymorphisms demonstrated a nominally significant association with outcome, although findings did not reach significance after correcting for multiple testing.^57^

The suggestive probe with the largest effect size at follow-up was part of the high-affinity IgE receptor *FCER1G* gene, which is known to be a key unit in allergic reactions and regulates several aspects of the immune response. Interestingly, this was not the only immune-related gene implicated in the top probes, with *IFI30* (associated with immune regulation and previously implicated in posttraumatic stress disorder; Neylan *et al.*^58^) and *STAT1* (regulation of immune related signalling; Hu and Ivashkiv^59^) both showing suggestive results. Previous research has implicated immune pathways as an important biological system involved in many mental health outcomes^60, 61, 62^ and as such this represents a plausible candidate for psychological therapy response.

Another interesting probe detected at follow-up was part of the *S100A10* gene, which encodes for the p11 protein. The gene is thought to play a role in serotonergic signalling through interaction with serotonin receptors, and has also been linked to response to stress via regulation by glucocorticoids (Svenningsson *et al.*^63^). Downregulation of p11 mRNA and protein levels has been implicated in depressive behaviours and risk of suicide, and several categories of antidepressants have been shown to increase p11 expression in brain tissues (Svenningsson *et al.*^63^). Conversely, in the sample presented here, a decrease in *S100A10* expression was observed corresponding with a better response to treatment. Also, it should be noted that gene expression in the current study was determined from peripheral blood samples (not brain regions). Nevertheless, while the role of this gene in response to psychological therapies is unclear, expression of the *S100A10* gene represents an interesting potential target for further research.

Previous studies examining candidate gene expression and response to a psychological therapy have predominantly focussed on the HPA-axis-related gene *FKBP5*.^16, 17^ Notably, gene expression changes in *FKBP5* were not associated with treatment outcome at post treatment in our sample, and were only nominally associated with outcome at follow-up. In fact, contrary to previous reports, participants with a better treatment outcome at follow-up displayed a reduction in *FKBP5* expression. However, there were some differences between these studies and the current report. Namely, previous research used a candidate gene approach, compared to the genome-wide approach utilised in the current study. Sample sizes were also smaller (*n*=39, *n*=20), and both studies focussed on adults with posttraumatic stress disorder rather than the range of anxiety diagnoses reported here.

As discussed in the Introduction, the majority of studies examining genetic and epigenetic correlates of response to psychological therapy currently also focus on candidate genes. The first genome-wide association study of therapy response (in children with anxiety disorders^64^) found no genetic variants significantly associated with psychological treatment outcome. In addition, previously implicated candidate genes (where available in the array data) were not replicated, suggesting that the effect of individual genes on treatment response is likely to be small. Studies of DNA methylation predictors of treatment response and changes during treatment have yielded moderate effect sizes, though to date few samples have more than 100 participants, and as yet no study has used epigenome-wide data in the context of psychological treatment response.

As well as the known mechanistic relationship between epigenetic patterns and gene expression, research has demonstrated that underlying genetic variation has the potential to influence both DNA methylation (for example, allele-specific methylation^65^) and gene expression (for example, eQTL^66^). Recent work examining factors associated with response to negative environmental influences (such as abuse) has suggested that epigenetic changes associated with psychological outcomes may be influenced by genotype.^67^ To this end, a study from our team showed genotype-dependent changes in *FKBP5* DNA methylation associated with treatment outcome.^14^ However, in a companion paper to this article combining genome-wide genetic and transcriptomic data (including an extended sample from this cohort^38^), a number of eQTLs (DNA sequence variation associated with gene expression) were identified, but interactions between genetic variants and treatment response were not significantly associated with expression levels. Nonetheless, future therapygenetics research would benefit from the continued use of genome-wide protocols, and the integration of genetic, epigenetic and transcriptomic information in order to elucidate the potential biological mechanisms underlying psychological treatment response.

In this study, we also performed data-driven clustering using WGCNA methods (detailed in the Supplementary Information^42^) but results from these analyses were not informative. Six network modules were identified within the pre-treatment data, but did not show any substantial changes in composition across the treatment period, indicating a largely similar molecular landscape across the treatment period. Changes in the expression profiles of these modules were not found to be associated with treatment outcome at post treatment or at follow-up. We also examined potential biological pathways by conducting enrichment analysis of the probe-level results. The results implicated that a number of functional processes such as cell cycle regulation were overrepresented in the top-ranked genes.

To our knowledge, this is the first study to examine gene expression profiles at a genome-wide level with respect to treatment outcome following a psychological therapy. However, the top results discussed here do not reach statistical significance, and should be considered preliminary. In addition, there are a number of caveats in the design that should be considered. Firstly, while the work presented here represents the largest study of gene expression with respect to psychological therapy outcomes, the sample size is still relatively small. Effect sizes for changes in symptoms are likely to be larger than for the corresponding changes in gene expression. Using the gene with the largest effect size in the suggestive results reported here as an example (FCER1G), statistical power analyses suggest that a sample size greater than 230 would be required to accurately detect an effect of the same size with 80% power (conducted using powerreg in Stata). Using a threshold of *α*=1.4 × 10-5 (reflecting the 4381 gene probes considered), the current sample was large enough to detect a variant explaining 23% of the variance in change in symptoms with 80% power (conducted using the pwr package in R). Statistical power is enhanced by the use of network-based approaches such as WGCNA. However, our sample was still only large enough to detect a module explaining 11% of the variance in change in symptoms with 80% power. Given that treatment response is thought to be influenced by a number of factors of small effect, it is likely that the current study is underpowered, and much greater sample sizes are needed. Linked to this issue, this sample had a moderate level of heterogeneity, with variability in treatment specifics (such as length) and diagnoses that may have affected the results. In addition, no control group (who had not received treatment) was available for this study. Therefore, it is possible that any changes detected in our sample may be explained by other factors. Future studies in this growing area would benefit from a focus on collecting much larger samples, preferably with control groups for comparison. The expansion of internet-based CBT trials and large CBT services, enabling therapists and treatment centres to reach a wider number of participants, are likely to be a useful resource for research examining predictors of treatment response. Greater sample sizes may also be achieved through large-scale collaborations, combining data from multiple treatment sites, treatment protocols and diagnoses. Although these approaches will likely lead to greater heterogeneity in the sample, for a predictor or biological correlate of treatment response to have a potential clinical utility it must be able to rise above this noise robustly in a range of treatment settings.

Secondly, in this study we have used clinician-rated severity of the treated diagnosis as the primary treatment outcome. Although the CGI is a well recognised and validated measure, it only concerns the severity of disorder. Another important aspect of treatment outcome in mental disorders, particularly in fear-related disorders such as anxiety, is reduction in impairment. Further work in this area should consider changes in levels of impairment as well as diagnosis severity. Finally, this study was conducted in a clinical setting with live participants, and therefore required the use of peripheral samples. However, whilst peripheral samples may not be wholly representative of brain expression patterns, transcriptomic comparisons in previous research have indicated that the expression of over 80% of genes is shared between peripheral blood and other tissues (including brain).^68^ In addition, the use of peripheral tissue is a necessity of biomarker and treatment response studies.

## Conclusion

In summary, this study represents the first report of genome-wide expression profiles with respect to response to a psychological therapy. We find suggestive evidence for the role of gene expression changes in a small number of biologically plausible candidate genes, although none reach a corrected level of significance. If replicated, these findings have the potential to further our understanding of the biological changes underlying response to psychological therapies.

## Figures and Tables

**Table 1:** Associations between percentage change in CGI-S and clinical factors

*Clinical factor*	*Percentage change in CGI-S*			
	*Post treatment*		*Follow-up*	
	r	P	r	P
Baseline severity	−0.051	0.616	0.148	0.157
Age	−0.120	0.234	−0.015	0.887
BMI	0.010	0.921	−0.003	0.976

	t(df)	P	t(df)	P
Gender	−0.913 (98)	0.364	−0.104 (91)	0.917
Smoking status	0.390 (98)	0.698	0.500 (91)	0.618
Comorbidities	1.126 (98)	0.263	1.120 (91)	0.235
Psychoactive medication	3.498 (98)	7E−04*	2.287 (91)	0.025*
Other medications	1.266 (98)	0.209	0.823 (91)	0.413

Population stratification	r	P	r	P
PC1	−0.178	0.082	−0.003	0.981
PC2	−0.078	0.451	0.033	0.758

Cell proportion changes	r	P	r	P
*Lymphocytes*				
Pre- to post treatment	0.047	0.657	—	—
Pre-treatment to follow-up	—	—	0.079	0.481
				
*Neutrophils*				
Pre- to post treatment	0.040	0.710	—	—
Pre-treatment to follow-up	—	—	0.040	0.720
				
*Monocytes*				
Pre- to post treatment	0.030	0.780	—	—
Pre-treatment to follow-up	—	—	−0.097	0.399

Abbreviation: BMI, body mass index; CGI-S, Clinical Global Impression—Severity. NB: factors that were nominally significant (*) at either time point were included as covariates in linear-mixed models.

**Table 2:** Top genes ranked by association with treatment outcome (percentage improvement in CGI-S)

*Time*	*Gene symbol*	*Probe ID*	*Test* β	P*-value*	q*-value*
Post treatment	**ATP5D**	**ILMN_1653599**	**−0.373**	**8.90E−05**	**0.353**
	HBA2	ILMN_2127842	0.670	3.87E−04	0.767
	ATP5H	ILMN_1794912	−0.795	8.12E−04	0.890
	LOC646630	ILMN_1691449	−0.588	1.06E−03	0.890
	HSP90AB1	ILMN_1673711	−0.543	1.90E−03	0.890
	TMEM160	ILMN_1704024	−0.609	2.37E−03	0.890
	NT5C3	ILMN_2352121	−0.204	2.57E−03	0.890
	RPL23A	ILMN_1788607	−0.960	2.84E−03	0.890
	F2R	ILMN_2221507	0.496	4.06E−03	0.890
	NDUFA12	ILMN_1737738	−0.885	4.77E−03	0.890

Follow-up	**PTBP1**	**ILMN_2333319**	**0.715**	**4.11E−04**	**0.499**
	**DAGLB**	**ILMN_1658885**	**0.713**	**6.02E−04**	**0.499**
	**FCER1G**	**ILMN_2123743**	**−1.176**	**6.33E−04**	**0.499**
	**LOC647506**	**ILMN_3240375**	**0.228**	**6.65E−04**	**0.499**
	**LOC652493**	**ILMN_1739508**	**0.184**	**1.18E−03**	**0.499**
	**IFI30**	**ILMN_1807277**	**−0.677**	**1.12E−03**	**0.499**
	**S100A10**	**ILMN_1796712**	**−0.428**	**1.24E−03**	**0.499**
	**LOC642113**	**ILMN_1652199**	**0.198**	**1.25E−03**	**0.499**
	**ACP1**	**ILMN_2344956**	**−0.633**	**1.59E−03**	**0.499**
	**SPCS2**	**ILMN_1809488**	**−0.547**	**1.69E−03**	**0.499**
	**SRRM1**	**ILMN_1697670**	**0.705**	**1.74E−03**	**0.499**
	**PIK3R1**	**ILMN_1760303**	**0.808**	**1.90E−03**	**0.499**
	**HNRPA1P4**	**ILMN_1690586**	**0.562**	**2.10E−03**	**0.499**
	**STUB1**	**ILMN_1756126**	**−0.429**	**2.10E−03**	**0.499**
	**PCM1**	**ILMN_1690487**	**0.869**	**2.34E−03**	**0.499**
	**OCIAD1**	**ILMN_1799604**	**0.462**	**2.36E−03**	**0.499**
	**PSMB10**	**ILMN_1683026**	**−0.685**	**2.38E−03**	**0.499**
	**STAT1**	**ILMN_1691364**	**−0.230**	**2.48E−03**	**0.499**

Abbreviation: CGI-S, Clinical Global Impression—Severity. NB: probe ID=Illumina HumanHT12 v4 expression BeadChip array ID, ‘Test *β*’=test statistic for association with treatment outcome, values in bold indicates probes significant at a suggestive level.
