# Supplementary material for: Genome-wide expression and response to exposure-based psychological therapy for anxiety disorders
Source: Transl Psychiatry. 2017 Aug 29;7(8):e1219–. doi: 10.1038/tp.2017.177 (PMC5611743; doi:10.1038/tp.2017.177)
Supplement: Supplementary Information [file tp2017177x1.docx]

**Supplementary Information**

*Treatment protocols and sample information*

**Biological Pathway Enrichment Analyses**

GOrilla identifies enrichment in ranked lists of genes by calculating a minimum hypergeometric score. The ranked list is split into a target set (the first *x* genes) and the background set (the entire list). This is performed iteratively, adding each gene into the target set until all genes are included. The enrichment score is calculated from the optimal target set, using a hypergeometric distribution, correcting for the multiple thresholding involved in the method (Eden et al., 2009).

**Network-based analyses**

Co-expression network analysis using Weighted Gene Correlation Network Analysis (WGCNA) is a powerful approach, even in relatively small datasets, as it describes the correlation patterns among genes and therefore reduces the multiple testing burden associated with genome-wide data.

*WGCNA*

Data-driven clustering was performed using WGCNA on pre-treatment samples to create signed co-expression networks (Langfelder and Horvath, 2008). Modules detected at pre-treatment were forced onto the data at the post-treatment and follow-up time-points using the module preservation function (Langfelder et al.) in order to examine changes across the course of treatment.

Module eigengenes, equivalent to the first principal component of each module, were used as a proxy for module expression. The association between change in CGI-S severity and change in module expression was tested using linear regression models for post-treatment and follow-up as previously described. The grey module was not included in any analyses as it consisted of genes that were unable to be assigned to a module.

*Results*

Six co-expression modules were detected at pre-treatment (excluding the grey module). Modules were well preserved at post-treatment and follow-up (module details can be found in the supplementary information). No significant association was found between percentage reduction in CGI-S severity and change in module eigengene for any module at post-treatment (Table S3) or follow-up (Table S4).

*Overview*

Six network modules were identified within the pre-treatment data, but did not show any substantial changes in composition across the treatment period. Changes in the expression profiles of these modules were not found to be associated with treatment outcome at post-treatment or at follow-up.

**Table S1 Top 100 ranked probes associated with treatment outcome at post-treatment**

| Symbol | probeID | Test β | *p*-value |
| --- | --- | --- | --- |
| ATP5D | ILMN_1653599 | -.3737943 | .000089 |
| HBA2 | ILMN_2127842 | .6697792 | .0003867 |
| ATP5H | ILMN_1794912 | -.7949814 | .0008122 |
| LOC646630 | ILMN_1691449 | -.587799 | .0010579 |
| HSP90AB1 | ILMN_1673711 | -.5431891 | .0019024 |
| TMEM160 | ILMN_1704024 | -.6094695 | .0023687 |
| NT5C3 | ILMN_2352121 | -.2040663 | .002567 |
| RPL23A | ILMN_1788607 | -.9604254 | .0028394 |
| F2R | ILMN_2221507 | .4963429 | .004058 |
| NDUFA12 | ILMN_1737738 | -.8853866 | .0047687 |
| CCDC56 | ILMN_2064898 | -.8079423 | .0049181 |
| TNFSF12 | ILMN_1683700 | -.9698006 | .0049981 |
| SCYL1 | ILMN_2400874 | .8029177 | .0056379 |
| LOC728553 | ILMN_3299407 | -.3281274 | .0058962 |
| CKLF | ILMN_2414027 | -.163631 | .0070256 |
| BANF1 | ILMN_2179837 | -.6652999 | .0078082 |
| MRPL18 | ILMN_2230672 | -.5548399 | .0079131 |
| TMEM93 | ILMN_1758674 | -.4645427 | .0083014 |
| C15orf24 | ILMN_2150000 | -1.015606 | .0084504 |
| TPST2 | ILMN_2329679 | -.4628873 | .0085511 |
| NDUFA11 | ILMN_2175712 | -.3863854 | .0091398 |
| TIMM23 | ILMN_1679555 | -.7375876 | .0092518 |
| RPA2 | ILMN_1753582 | -.7737793 | .0093347 |
| CLUAP1 | ILMN_2242491 | .6376041 | .0093829 |
| GAPDH | ILMN_1802252 | -.2039513 | .0098436 |
| PSME1 | ILMN_1806017 | -.5540774 | .0099316 |
| TMBIM4 | ILMN_1664750 | .2945278 | .0105145 |
| OSTC | ILMN_1776005 | -.3188746 | .0107912 |
| HBA2 | ILMN_1667796 | .6114666 | .0108061 |
| PPM1B | ILMN_1716862 | -.1027366 | .0108376 |
| PPP1CB | ILMN_2405018 | -.1081466 | .0119464 |
| ADD3 | ILMN_2311278 | .2012233 | .0120307 |
| FAM177A1 | ILMN_2394438 | .9968996 | .0120965 |
| LOC100133876 | ILMN_3202483 | .6625076 | .0123497 |
| CXXC5 | ILMN_1745256 | .3837985 | .0123891 |
| HBA1 | ILMN_3240144 | .8039176 | .0125179 |
| GAPT | ILMN_3242271 | -.3801056 | .0134256 |
| OAZ1 | ILMN_1773080 | .6049531 | .0147815 |
| IFI6 | ILMN_2347798 | -.2347861 | .0150835 |
| ATG7 | ILMN_1790978 | .1989745 | .0154187 |
| AMD1 | ILMN_1788462 | -.3280054 | .0154687 |
| LPIN2 | ILMN_1670028 | .3278612 | .0155696 |
| EIF4G2 | ILMN_2380946 | .3064855 | .0156793 |
| GUSBL1 | ILMN_1720857 | .3745367 | .0160086 |
| CGGBP1 | ILMN_2387090 | .5185171 | .0160259 |
| SF3B14 | ILMN_1703720 | -.2893094 | .016795 |
| LOC647285 | ILMN_3289100 | -.470793 | .0175217 |
| FPR1 | ILMN_2092118 | .2492087 | .0177706 |
| C19orf31 | ILMN_2233279 | .6588982 | .018103 |
| RPS2 | ILMN_2218277 | -.6245843 | .0186021 |
| IMAA | ILMN_2234515 | .5655854 | .0188564 |
| MX1 | ILMN_1662358 | -.1153901 | .0190801 |
| LOC441743 | ILMN_2110829 | -.5577753 | .0192029 |
| LOC391019 | ILMN_3286813 | -.4450042 | .0192127 |
| COX7A2 | ILMN_1701293 | -.3901424 | .0195177 |
| C22orf30 | ILMN_3307887 | .6671384 | .0195305 |
| EIF5A | ILMN_1794522 | -.3888165 | .0201102 |
| ROMO1 | ILMN_3234993 | -.3000958 | .0202299 |
| MED28 | ILMN_1749821 | -.4616364 | .0202519 |
| FAM129A | ILMN_1810725 | .2937445 | .0204947 |
| BOLA2 | ILMN_1782633 | -.4916711 | .0206993 |
| GARNL4 | ILMN_2041236 | .3530984 | .0209573 |
| LOC100131905 | ILMN_3275489 | -.1439108 | .0210018 |
| ZC3H11B | ILMN_3303965 | .3895602 | .0210053 |
| RANBP1 | ILMN_2109156 | -.7886558 | .0210337 |
| RANGAP1 | ILMN_1662198 | -.2060024 | .0211432 |
| SSU72 | ILMN_1664956 | -.3629505 | .0213559 |
| KIAA0430 | ILMN_1793371 | .3735335 | .021785 |
| HLA-E | ILMN_1765258 | .4787273 | .022769 |
| LOC148430 | ILMN_3278506 | -.3575286 | .0228927 |
| KRTCAP2 | ILMN_1658802 | -.6024988 | .0233779 |
| PSMA3 | ILMN_2387553 | -.5631199 | .0235401 |
| COASY | ILMN_1753498 | .6853045 | .0239516 |
| RAB11A | ILMN_1712312 | -.3065159 | .0239582 |
| AKR7A2 | ILMN_1677043 | -.7192659 | .0240816 |
| OSBPL8 | ILMN_1782459 | -.0707115 | .0244391 |
| DEFA1B | ILMN_1679357 | .2524741 | .0246268 |
| GBP5 | ILMN_2114568 | .2593783 | .0248784 |
| HBB | ILMN_2100437 | .5749836 | .0249061 |
| MGC10997 | ILMN_2147251 | .6565144 | .0249512 |
| KIF22 | ILMN_3234884 | -.6659959 | .0249552 |
| LOC643873 | ILMN_3275672 | -.1708477 | .0252182 |
| EIF3B | ILMN_2379469 | .632057 | .0252357 |
| ITGB2 | ILMN_2175912 | .6764562 | .0252731 |
| YY1AP1 | ILMN_1752303 | .1344282 | .0256226 |
| CD44 | ILMN_1803429 | .6252132 | .0264368 |
| UBL5 | ILMN_2340065 | -.3468317 | .0267602 |
| LOC100133465 | ILMN_3243549 | .7494463 | .0269712 |
| EDF1 | ILMN_2246894 | -.8966335 | .0270524 |
| DDT | ILMN_1690982 | -.7610465 | .0272007 |
| COMMD1 | ILMN_1761242 | -.6987549 | .0276195 |
| PTPRCAP | ILMN_1672417 | -.3049896 | .0279428 |
| FLJ22662 | ILMN_1707286 | .2273323 | .0280437 |
| GLRX | ILMN_1737308 | -.2328231 | .0284907 |
| LOC100130003 | ILMN_3256004 | -.1731221 | .028859 |
| CD1D | ILMN_1719433 | .3989671 | .0294393 |
| BTG2 | ILMN_1770085 | .4090464 | .0294656 |
| LOC645173 | ILMN_3211132 | -.5087918 | .0295482 |
| LOC132241 | ILMN_1701247 | .6201155 | .0298833 |
| PAPOLA | ILMN_1798354 | .1787079 | .0299781 |

**Table S2 Top pathways in post-treatment results identified using GOrilla**

| GO Term | Description | P-value | FDR q-value | Enrichment | no. of genes |
| --- | --- | --- | --- | --- | --- |
| GO:0072599 | establishment of protein localization to endoplasmic reticulum | 3.51E-06 | 3.00E-02 | 1.84 | 52 |
| GO:0070972 | protein localization to endoplasmic reticulum | 4.68E-06 | 2.00E-02 | 1.82 | 53 |
| GO:0045047 | protein targeting to ER | 6.38E-06 | 1.82E-02 | 1.82 | 51 |
| GO:0006614 | SRP-dependent cotranslational protein targeting to membrane | 7.61E-06 | 1.63E-02 | 1.83 | 50 |
| GO:0006613 | cotranslational protein targeting to membrane | 1.15E-05 | 1.97E-02 | 1.81 | 50 |
| GO:0006413 | translational initiation | 1.53E-05 | 2.18E-02 | 1.68 | 63 |
| GO:0043624 | cellular protein complex disassembly | 2.60E-05 | 3.18E-02 | 1.74 | 53 |
| GO:0019083 | viral transcription | 3.53E-05 | 3.77E-02 | 1.84 | 43 |
| GO:0006414 | translational elongation | 4.00E-05 | 3.80E-02 | 1.7 | 54 |
| GO:0000184 | nuclear-transcribed mRNA catabolic process, nonsense-mediated decay | 8.64E-05 | 7.38E-02 | 1.74 | 48 |
| GO:0006415 | translational termination | 1.07E-04 | 8.35E-02 | 1.71 | 49 |
| GO:0043604 | amide biosynthetic process | 1.26E-04 | 8.99E-02 | 1.53 | 76 |
| GO:0006412 | translation | 1.31E-04 | 8.59E-02 | 1.54 | 73 |
| GO:0043043 | peptide biosynthetic process | 1.45E-04 | 8.85E-02 | 1.53 | 74 |
| GO:0044802 | single-organism membrane organization | 1.94E-04 | 1.11E-01 | 1.79 | 48 |
| GO:0006612 | protein targeting to membrane | 2.66E-04 | 1.42E-01 | 1.63 | 54 |
| GO:0043241 | protein complex disassembly | 3.79E-04 | 1.91E-01 | 1.61 | 54 |
| GO:0042776 | mitochondrial ATP synthesis coupled proton transport | 4.24E-04 | 2.01E-01 | 147.33 | 2 |
| GO:0006816 | calcium ion transport | 4.38E-04 | 1.97E-01 | 4.71 | 9 |
| GO:0035966 | response to topologically incorrect protein | 4.45E-04 | 1.90E-01 | 4.08 | 10 |
| GO:0000956 | nuclear-transcribed mRNA catabolic process | 5.05E-04 | 2.05E-01 | 1.58 | 56 |
| GO:0044765 | single-organism transport | 5.60E-04 | 2.18E-01 | 1.44 | 92 |
| GO:0019731 | antibacterial humoral response | 6.22E-04 | 2.31E-01 | 3.87 | 8 |
| GO:0006402 | mRNA catabolic process | 6.52E-04 | 2.32E-01 | 1.57 | 56 |
| GO:0032984 | macromolecular complex disassembly | 6.74E-04 | 2.30E-01 | 1.58 | 54 |
| GO:0015701 | bicarbonate transport | 6.97E-04 | 2.29E-01 | 24.17 | 3 |
| GO:0015985 | energy coupled proton transport, down electrochemical gradient | 7.12E-04 | 2.25E-01 | 121.33 | 2 |
| GO:0015986 | ATP synthesis coupled proton transport | 7.12E-04 | 2.17E-01 | 121.33 | 2 |
| GO:0044271 | cellular nitrogen compound biosynthetic process | 7.13E-04 | 2.10E-01 | 1.2 | 262 |
| GO:0019058 | viral life cycle | 7.13E-04 | 2.03E-01 | 2.21 | 26 |
| GO:1901566 | organonitrogen compound biosynthetic process | 7.58E-04 | 2.09E-01 | 1.34 | 114 |
| GO:0051234 | establishment of localization | 7.72E-04 | 2.06E-01 | 1.37 | 111 |
| GO:1902578 | single-organism localization | 7.85E-04 | 2.03E-01 | 1.42 | 95 |
| GO:0022904 | respiratory electron transport chain | 8.26E-04 | 2.08E-01 | 1.82 | 33 |
| GO:0022900 | electron transport chain | 8.26E-04 | 2.02E-01 | 1.82 | 33 |
| GO:0019438 | aromatic compound biosynthetic process | 8.43E-04 | 2.00E-01 | 1.21 | 231 |
| GO:0030518 | intracellular steroid hormone receptor signaling pathway | 9.17E-04 | 2.12E-01 | 4.8 | 8 |
| GO:0018130 | heterocycle biosynthetic process | 9.45E-04 | 2.13E-01 | 1.21 | 230 |

**Table S3 Top 100 ranked probes associated with treatment outcome at follow-up**

| Symbol | probeID | Test β | p-value |
| --- | --- | --- | --- |
| PTBP1 | ILMN_2333319 | .7148571 | .0004109 |
| DAGLB | ILMN_1658885 | .7128897 | .0006019 |
| FCER1G | ILMN_2123743 | -1.176274 | .000633 |
| LOC647506 | ILMN_3240375 | .2276649 | .0006649 |
| --- | ILMN_1821176 | .6114451 | .0009512 |
| LOC652493 | ILMN_1739508 | .1841703 | .0011837 |
| IFI30 | ILMN_1807277 | -.6770415 | .0012266 |
| S100A10 | ILMN_1796712 | -.4280953 | .0012357 |
| LOC642113 | ILMN_1652199 | .1978233 | .0012505 |
| ACP1 | ILMN_2344956 | -.6329149 | .0015947 |
| SPCS2 | ILMN_1809488 | -.5465101 | .0016851 |
| SRRM1 | ILMN_1697670 | .7052076 | .0017428 |
| PIK3R1 | ILMN_1760303 | .8075006 | .001898 |
| HNRPA1P4 | ILMN_1690586 | .5624513 | .0020961 |
| STUB1 | ILMN_1756126 | -.4289483 | .0021014 |
| PCM1 | ILMN_1690487 | .8687963 | .002341 |
| OCIAD1 | ILMN_1799604 | .4622046 | .0023595 |
| PSMB10 | ILMN_1683026 | -.684986 | .0023805 |
| STAT1 | ILMN_1691364 | -.2296256 | .0024793 |
| SDHB | ILMN_1667257 | -1.677594 | .0029947 |
| NUP62 | ILMN_1738681 | .5268869 | .0030605 |
| --- | ILMN_1841334 | .7512956 | .0031761 |
| LPIN1 | ILMN_1671554 | .5563229 | .0032578 |
| PRDX5 | ILMN_1711606 | -.8970807 | .003615 |
| RAB32 | ILMN_2115434 | -.6929277 | .003902 |
| AIF1 | ILMN_1703538 | -1.060049 | .0039728 |
| ITGB1 | ILMN_2383934 | .6033691 | .0042105 |
| IFI35 | ILMN_1745374 | -.541114 | .0045016 |
| BCL2 | ILMN_1801119 | .4038037 | .0045054 |
| NDUFA13 | ILMN_1767139 | -1.116936 | .0045721 |
| DRAP1 | ILMN_2112301 | -.8108366 | .0047144 |
| COX8A | ILMN_1809495 | -.9511845 | .0047863 |
| PURB | ILMN_1750079 | .7541142 | .0047901 |
| SYPL1 | ILMN_1764087 | .6453888 | .0049082 |
| PSME2 | ILMN_1786612 | -.7156108 | .0049354 |
| CAT | ILMN_1651705 | -.3792795 | .0052954 |
| COX6B1 | ILMN_2154671 | -2.217585 | .0053559 |
| AP2S1 | ILMN_1809957 | -.6443163 | .0056663 |
| SASH3 | ILMN_1697554 | -.5317902 | .0056792 |
| CCM2 | ILMN_1784352 | .7704736 | .005831 |
| WARS | ILMN_1727271 | -.4582743 | .0059705 |
| ATP8B2 | ILMN_1782057 | .9399057 | .0061588 |
| LOC402251 | ILMN_1687080 | -.1128917 | .0063756 |
| SRP54 | ILMN_2312275 | 1.006286 | .0066782 |
| PSMA1 | ILMN_1709611 | -.8901752 | .0068024 |
| TROVE2 | ILMN_2311518 | .6205201 | .006817 |
| CARD11 | ILMN_1721978 | 1.058289 | .0076836 |
| TMEM219 | ILMN_1737644 | -.8937208 | .0078 |
| GLT25D1 | ILMN_1727043 | .3066821 | .0078239 |
| PSMB8 | ILMN_1747195 | -.8742238 | .0079862 |
| TRIM38 | ILMN_1697971 | .3453327 | .0083563 |
| COX5B | ILMN_1663512 | -1.812507 | .0084375 |
| ARHGEF6 | ILMN_1803423 | 1.204583 | .0084684 |
| C19orf43 | ILMN_1784299 | -.9052611 | .0085036 |
| LOC402175 | ILMN_3291709 | -1.008311 | .0087558 |
| NDUFA12 | ILMN_1737738 | -.8828268 | .0091021 |
| OGT | ILMN_2364828 | .5749437 | .00947 |
| TOP2B | ILMN_1777663 | 1.196825 | .0100015 |
| ZFYVE20 | ILMN_1799890 | 1.092933 | .0101455 |
| TMEM183B | ILMN_2172269 | -.4779046 | .0105932 |
| HLA-A | ILMN_1671054 | -.7063101 | .0106829 |
| RPS26 | ILMN_2209027 | -.5526984 | .0109409 |
| LOC642333 | ILMN_1696027 | .7002961 | .0109935 |
| CCDC23 | ILMN_2050023 | -.6005231 | .0109973 |
| CCND2 | ILMN_2067656 | .8002707 | .0110958 |
| SFRS5 | ILMN_1761996 | 1.518095 | .0112283 |
| NUB1 | ILMN_1665291 | -.4090649 | .0116228 |
| RNF181 | ILMN_1655340 | -1.129382 | .0116278 |
| ETS1 | ILMN_2122103 | .6007917 | .0119479 |
| FAF2 | ILMN_1670472 | 1.359654 | .012075 |
| PSTPIP1 | ILMN_1703327 | .5355247 | .0122227 |
| ILK | ILMN_2364376 | -1.129931 | .0123087 |
| AIF1 | ILMN_1792473 | -.8610772 | .0130073 |
| NIPA2 | ILMN_1720344 | 1.141479 | .0130758 |
| BCL11B | ILMN_1665761 | .8883782 | .013388 |
| SF3B14 | ILMN_1703720 | -.6036161 | .0134556 |
| LOC100131609 | ILMN_3292224 | .3241105 | .0134946 |
| LOC100128266 | ILMN_3260070 | -.6060733 | .0136775 |
| BLOC1S1 | ILMN_2157510 | -.814507 | .0141277 |
| GLOD4 | ILMN_1738656 | .7663223 | .0146774 |
| LOC728666 | ILMN_3300744 | -1.016329 | .0152051 |
| LTB | ILMN_2376205 | .7581202 | .015373 |
| SYT11 | ILMN_1717934 | .8440681 | .015554 |
| C5orf41 | ILMN_1776788 | .5997579 | .0155782 |
| GRK5 | ILMN_2096719 | 1.175988 | .0156556 |
| MSH6 | ILMN_1729051 | .7232942 | .0159653 |
| LOC100131801 | ILMN_3209832 | -.7518864 | .0162194 |
| EWSR1 | ILMN_1727041 | .9124303 | .016487 |
| HSD17B12 | ILMN_2094106 | .6789533 | .0171413 |
| HNRPC | ILMN_1695588 | -.3723766 | .0173544 |
| ATXN7L3 | ILMN_1862018 | -.5995508 | .0175618 |
| TXN | ILMN_2038776 | -.6374302 | .0176267 |
| MTMR11 | ILMN_1769299 | -.4704068 | .0180066 |
| TMEM149 | ILMN_1786426 | -.4539388 | .0181903 |
| LPP | ILMN_1651254 | .72497 | .018865 |
| PKM2 | ILMN_2366634 | -.3161919 | .019208 |
| ATHL1 | ILMN_1794707 | .5238816 | .019299 |
| SSR2 | ILMN_1783226 | -.2802047 | .0195252 |
| NDUFB8 | ILMN_1661170 | -.7100343 | .0196154 |
| PSME1 | ILMN_1806017 | -.6709799 | .0197784 |

**Table S4 Top pathways in follow-up results identified using GOrilla**

| GO Term | Description | P-value | FDR q-value | Enrichment | No. of genes |
| --- | --- | --- | --- | --- | --- |
| GO:0051437 | positive regulation of ubiquitin-protein ligase activity involved in regulation of mitotic cell cycle transition | 1.47E-05 | 5.00E-03 | 2.54 | 23 |
| GO:0072422 | signal transduction involved in DNA damage checkpoint | 1.47E-05 | 6.00E-03 | 2.54 | 23 |
| GO:0072413 | signal transduction involved in mitotic cell cycle checkpoint | 1.47E-05 | 6.00E-03 | 2.54 | 23 |
| GO:0072401 | signal transduction involved in DNA integrity checkpoint | 1.47E-05 | 6.00E-03 | 2.54 | 23 |
| GO:0043067 | regulation of programmed cell death | 1.61E-05 | 6.00E-03 | 1.87 | 55 |
| GO:1901988 | negative regulation of cell cycle phase transition | 1.71E-05 | 6.00E-03 | 2.13 | 34 |
| GO:1902807 | negative regulation of cell cycle G1/S phase transition | 1.77E-05 | 6.00E-03 | 2.34 | 27 |
| GO:2000134 | negative regulation of G1/S transition of mitotic cell cycle | 1.77E-05 | 6.00E-03 | 2.34 | 27 |
| GO:0010498 | proteasomal protein catabolic process | 1.89E-05 | 6.00E-03 | 3.18 | 21 |
| GO:0043161 | proteasome-mediated ubiquitin-dependent protein catabolic process | 1.89E-05 | 6.00E-03 | 3.18 | 21 |
| GO:2000058 | regulation of protein ubiquitination involved in ubiquitin-dependent protein catabolic process | 2.25E-05 | 6.00E-03 | 2.45 | 24 |
| GO:2000060 | positive regulation of protein ubiquitination involved in ubiquitin-dependent protein catabolic process | 2.25E-05 | 6.00E-03 | 2.45 | 24 |
| GO:1901987 | regulation of cell cycle phase transition | 7.24E-06 | 7.00E-03 | 2.11 | 39 |
| GO:0006977 | DNA damage response, signal transduction by p53 class mediator resulting in cell cycle arrest | 1.47E-05 | 7.00E-03 | 2.54 | 23 |
| GO:0072395 | signal transduction involved in cell cycle checkpoint | 1.47E-05 | 7.00E-03 | 2.54 | 23 |
| GO:0072431 | signal transduction involved in mitotic G1 DNA damage checkpoint | 1.47E-05 | 7.00E-03 | 2.54 | 23 |
| GO:0051352 | negative regulation of ligase activity | 2.61E-05 | 7.00E-03 | 2.23 | 27 |
| GO:0051444 | negative regulation of ubiquitin-protein transferase activity | 2.61E-05 | 7.00E-03 | 2.23 | 27 |
| GO:0009968 | negative regulation of signal transduction | 2.92E-05 | 7.00E-03 | 2.12 | 39 |
| GO:0070647 | protein modification by small protein conjugation or removal | 3.05E-05 | 7.00E-03 | 2.3 | 35 |
| GO:0043069 | negative regulation of programmed cell death | 5.85E-06 | 8.00E-03 | 2.93 | 25 |
| GO:0051436 | negative regulation of ubiquitin-protein ligase activity involved in mitotic cell cycle | 6.82E-06 | 8.00E-03 | 2.34 | 27 |
| GO:0048585 | negative regulation of response to stimulus | 7.06E-06 | 8.00E-03 | 2.05 | 47 |
| GO:1901796 | regulation of signal transduction by p53 class mediator | 9.75E-06 | 8.00E-03 | 3.42 | 11 |
| GO:1902403 | signal transduction involved in mitotic DNA integrity checkpoint | 1.47E-05 | 8.00E-03 | 2.54 | 23 |
| GO:1902402 | signal transduction involved in mitotic DNA damage checkpoint | 1.47E-05 | 8.00E-03 | 2.54 | 23 |
| GO:1901990 | regulation of mitotic cell cycle phase transition | 3.19E-05 | 8.00E-03 | 2.05 | 37 |
| GO:1901991 | negative regulation of mitotic cell cycle phase transition | 3.48E-05 | 8.00E-03 | 2.1 | 33 |
| GO:0051439 | regulation of ubiquitin-protein ligase activity involved in mitotic cell cycle | 1.17E-05 | 9.00E-03 | 2.26 | 28 |
| GO:0010564 | regulation of cell cycle process | 1.33E-05 | 9.00E-03 | 1.81 | 55 |
| GO:0042981 | regulation of apoptotic process | 1.35E-05 | 9.00E-03 | 1.88 | 55 |
| GO:1902400 | intracellular signal transduction involved in G1 DNA damage checkpoint | 1.47E-05 | 9.00E-03 | 2.54 | 23 |
| GO:0023057 | negative regulation of signaling | 3.86E-05 | 9.00E-03 | 2.08 | 40 |
| GO:0010941 | regulation of cell death | 4.04E-05 | 9.00E-03 | 1.86 | 51 |
| GO:0000082 | G1/S transition of mitotic cell cycle | 5.84E-06 | 1.00E-02 | 2.37 | 29 |
| GO:0033554 | cellular response to stress | 4.61E-05 | 1.00E-02 | 1.89 | 49 |
| GO:0009411 | response to UV | 4.73E-05 | 1.00E-02 | 3.66 | 14 |
| GO:1902806 | regulation of cell cycle G1/S phase transition | 5.63E-05 | 1.20E-02 | 2.15 | 30 |
| GO:0045787 | positive regulation of cell cycle | 6.21E-05 | 1.20E-02 | 1.94 | 39 |
| GO:0042590 | antigen processing and presentation of exogenous peptide antigen via MHC class I | 6.26E-05 | 1.20E-02 | 2.2 | 28 |
| GO:0072331 | signal transduction by p53 class mediator | 6.26E-05 | 1.20E-02 | 2.2 | 28 |
| GO:0030330 | DNA damage response, signal transduction by p53 class mediator | 7.20E-05 | 1.20E-02 | 2.33 | 24 |
| GO:2000045 | regulation of G1/S transition of mitotic cell cycle | 7.37E-05 | 1.20E-02 | 2.15 | 29 |
| GO:0043632 | modification-dependent macromolecule catabolic process | 7.41E-05 | 1.20E-02 | 2.71 | 24 |
| GO:0044843 | cell cycle G1/S phase transition | 5.84E-06 | 1.30E-02 | 2.37 | 29 |
| GO:0002223 | stimulatory C-type lectin receptor signaling pathway | 6.71E-05 | 1.30E-02 | 1.94 | 35 |
| GO:0010648 | negative regulation of cell communication | 6.86E-05 | 1.30E-02 | 2.03 | 40 |
| GO:0031145 | anaphase-promoting complex-dependent proteasomal ubiquitin-dependent protein catabolic process | 7.06E-05 | 1.30E-02 | 2.43 | 22 |
| GO:0071156 | regulation of cell cycle arrest | 7.16E-05 | 1.30E-02 | 2.26 | 26 |
| GO:0006511 | ubiquitin-dependent protein catabolic process | 8.05E-05 | 1.30E-02 | 2.76 | 23 |
| GO:0022904 | respiratory electron transport chain | 9.07E-05 | 1.40E-02 | 1.98 | 34 |
| GO:0022900 | electron transport chain | 9.07E-05 | 1.40E-02 | 1.98 | 34 |
| GO:0032446 | protein modification by small protein conjugation | 9.62E-05 | 1.40E-02 | 2.28 | 32 |
| GO:0043516 | regulation of DNA damage response, signal transduction by p53 class mediator | 9.57E-05 | 1.50E-02 | 4.72 | 7 |
| GO:0010948 | negative regulation of cell cycle process | 5.64E-06 | 1.60E-02 | 2.09 | 39 |
| GO:0010467 | gene expression | 1.15E-04 | 1.70E-02 | 2.54 | 24 |
| GO:1901797 | negative regulation of signal transduction by p53 class mediator | 1.17E-04 | 1.70E-02 | 5.73 | 6 |
| GO:0051443 | positive regulation of ubiquitin-protein transferase activity | 1.21E-04 | 1.70E-02 | 2.28 | 24 |
| GO:0031397 | negative regulation of protein ubiquitination | 1.26E-04 | 1.70E-02 | 2 | 31 |
| GO:0090068 | positive regulation of cell cycle process | 1.25E-04 | 1.80E-02 | 2.01 | 33 |
| GO:0000209 | protein polyubiquitination | 1.32E-04 | 1.80E-02 | 3.87 | 14 |
| GO:0045930 | negative regulation of mitotic cell cycle | 1.35E-04 | 1.80E-02 | 2.01 | 34 |
| GO:0051603 | proteolysis involved in cellular protein catabolic process | 1.51E-04 | 2.00E-02 | 2.6 | 24 |
| GO:0042770 | signal transduction in response to DNA damage | 1.51E-04 | 2.00E-02 | 2.22 | 25 |
| GO:0019941 | modification-dependent protein catabolic process | 1.56E-04 | 2.00E-02 | 2.66 | 23 |
| GO:0030163 | protein catabolic process | 1.58E-04 | 2.00E-02 | 2.8 | 21 |
| GO:1903321 | negative regulation of protein modification by small protein conjugation or removal | 1.64E-04 | 2.00E-02 | 1.96 | 32 |
| GO:0071158 | positive regulation of cell cycle arrest | 1.65E-04 | 2.00E-02 | 2.29 | 23 |
| GO:0044772 | mitotic cell cycle phase transition | 1.66E-04 | 2.00E-02 | 1.89 | 38 |
| GO:0002479 | antigen processing and presentation of exogenous peptide antigen via MHC class I, TAP-dependent | 1.82E-04 | 2.10E-02 | 2.16 | 26 |
| GO:0002220 | innate immune response activating cell surface receptor signaling pathway | 1.82E-04 | 2.20E-02 | 1.88 | 35 |
| GO:0060548 | negative regulation of cell death | 5.37E-06 | 2.30E-02 | 2.88 | 26 |
| GO:0002474 | antigen processing and presentation of peptide antigen via MHC class I | 1.98E-04 | 2.30E-02 | 2 | 31 |
| GO:0006974 | cellular response to DNA damage stimulus | 2.11E-04 | 2.40E-02 | 1.73 | 54 |
| GO:0051351 | positive regulation of ligase activity | 2.12E-04 | 2.40E-02 | 2.22 | 24 |
| GO:0031398 | positive regulation of protein ubiquitination | 2.28E-04 | 2.50E-02 | 2.01 | 31 |
| GO:0044770 | cell cycle phase transition | 2.30E-04 | 2.50E-02 | 1.87 | 38 |
| GO:0001658 | branching involved in ureteric bud morphogenesis | 2.35E-04 | 2.50E-02 | 26.72 | 3 |
| GO:1903322 | positive regulation of protein modification by small protein conjugation or removal | 2.47E-04 | 2.60E-02 | 1.98 | 32 |
| GO:0035872 | nucleotide-binding domain, leucine rich repeat containing receptor signaling pathway | 2.80E-04 | 3.00E-02 | 3.14 | 12 |
| GO:0016567 | protein ubiquitination | 2.85E-04 | 3.00E-02 | 2.31 | 28 |
| GO:0060828 | regulation of canonical Wnt signaling pathway | 2.91E-04 | 3.00E-02 | 2.05 | 28 |
| GO:0014911 | positive regulation of smooth muscle cell migration | 2.93E-04 | 3.00E-02 | 89.86 | 2 |
| GO:0014854 | response to inactivity | 2.97E-04 | 3.00E-02 | 24.47 | 3 |
| GO:0090090 | negative regulation of canonical Wnt signaling pathway | 3.48E-04 | 3.50E-02 | 2.16 | 24 |
| GO:0090263 | positive regulation of canonical Wnt signaling pathway | 3.62E-04 | 3.60E-02 | 2.24 | 22 |
| GO:0019882 | antigen processing and presentation | 4.34E-04 | 4.20E-02 | 5.42 | 9 |
| GO:0043066 | negative regulation of apoptotic process | 5.07E-06 | 4.30E-02 | 2.96 | 25 |
| GO:0016265 | death | 4.85E-04 | 4.60E-02 | 2.26 | 26 |
| GO:0008219 | cell death | 4.85E-04 | 4.70E-02 | 2.26 | 26 |
| GO:0051438 | regulation of ubiquitin-protein transferase activity | 5.05E-04 | 4.80E-02 | 2.04 | 27 |

|  | Brown | Yellow | Turquoise | Red | Blue | Green |
| --- | --- | --- | --- | --- | --- | --- |

**Table S5. Change in CGI-S severity and change in module expression from pre- to post-treatment**

|  | β | CI | P | β | CI | p | β | CI | p | β | CI | p | β | CI | p | β | CI | p |
| --- | --- | --- | --- | --- | --- | --- | --- | --- | --- | --- | --- | --- | --- | --- | --- | --- | --- | --- |
| Module | 0.60 | -0.91 -2.11 | 0.294 | 0.22 | -0.28 -0.73 | 0.255 | -0.39 | -2.50 -1.72 | 0.601 | -0.45 | -5.18 -4.28 | 0.781 | -0.22 | -0.87 -0.43 | 0.355 | -1.14 | -3.55 -1.27 | 0.228 |
| Time | 0 | -0.00 - 0.00 | 0.849 | 0 | -0.00 -0.00 | 0.829 | 0 | -0.00 -0.00 | 0.842 | 0 | -0.00 -0.00 | 0.831 | 0 | -0.00 -0.00 | 0.848 | 0 | -0.00 -0.00 | 0.887 |
| Sessions | -0.01 | -0.10 - 0.09 | 0.801 | -0.01 | -0.10-0.08 | 0.816 | -0.01 | -0.10-0.09 | 0.822 | -0.01 | -0.09-0.08 | 0.829 | -0.01 | -0.10-0.08 | 0.807 | -0.01 | -0.09 -0.06 | 0.650 |
| Psycho- active medication | -1.64 | -1.95 - -1.32 | <0.001 | -1.67 | -2.01 - -1.33 | 0.001 | -1.65 | -1.96 - -1.33 | <0.001 | -1.67 | -2.05 - -1.28 | 0.001 | -1.67 | -1.93 - -1.40 | <0.001 | -1.67 | -1.98 - -1.36 | <0.001 |

**Table S6. Change in CGI-S severity and change in module expression from pre-treatment to follow-up**

|  | | | Β | CI | p | Β | CI | p | β | CI | p | β | CI | P | β | CI | p | β | CI | p |
| --- | --- | --- | --- | --- | --- | --- | --- | --- | --- | --- | --- | --- | --- | --- | --- | --- | --- | --- | --- | --- |
| Module | 0.98 | | | -2.85 -4.80 | 0.475 | -0.27 | -2.18 -1.64 | 0.68 | -1.01 | -6.37 -4.35 | 0.591 | -0.86 | -4.19 -2.46 | 0.469 | 0.12 | -2.06 -2.30 | 0.869 | -0.97 | -3.23 -1.28 | 0.262 |
| Time | | 0 | | -0.00 -0.00 | 0.51 | 0 | -0.00 -0.00 | 0.432 | 0 | -0.00 -0.00 | 0.567 | 0 | -0.00 -0.00 | 0.409 | 0 | -0.00 -0.00 | 0.434 | 0 | -0.00 -0.00 | 0.434 |
| Sessions | | 0 | | -0.07 -0.07 | 0.967 | 0 | -0.06 -0.06 | 0.991 | 0 | -0.08 -0.07 | 0.956 | 0 | -0.06 -0.05 | 0.934 | 0 | -0.06 -0.06 | 0.983 | 0 | -0.06 -0.06 | 0.864 |
| Psycho-active medication | | -1.02 | | -1.45 - -0.60 | 0.005 | -1.07 | -1.42 - -0.72 | 0.002 | -1.06 | -1.40 --0.71 | 0.002 | -1.15 | -1.63 --0.66 | 0.005 | -1.07 | -1.43 - -0.71 | 0.002 | -1.07 | -1.35 - -0.78 | 0.001 |

|  | Brown | Yellow | Turquoise | Red | Blue | Green |
| --- | --- | --- | --- | --- | --- | --- |

**References**

EDEN, E., NAVON, R., STEINFELD, I., LIPSON, D. & YAKHINI, Z. 2009. GOrilla: a tool for discovery and visualization of enriched GO terms in ranked gene lists. *BMC Bioinformatics,* 10**,** 48.

LANGFELDER, P. & HORVATH, S. 2008. WGCNA: an R package for weighted correlation network analysis. *BMC bioinformatics,* 9**,** 559.

LANGFELDER, P., LUO, R., OLDHAM, M. C. & HORVATH, S. Is my network module preserved and reproducible.
